# Obesity in Childhood and Adolescence: The Role of Motivation for Physical Activity, Self-Esteem, Implicit and Explicit Attitudes toward Obesity and Physical Activity

**DOI:** 10.3390/children10071177

**Published:** 2023-07-07

**Authors:** Silvia Scotto di Luzio, Guillaume Martinent, Maria Popa-Roch, Mathilde Ballereau, Soufyane Chahdi, Lucie Escudero, Emma Guillet-Descas

**Affiliations:** 1 Laboratoire Développement, Individu, Processus, Handicap, Éducation (DIPHE), Université Lyon 2, 69676 Bron, France; silvia.scotto-di-luzio@outlook.com; 2 Laboratoire sur les Vulnérabilités et L’innovation dans Le Sport (L-VIS), Université Claude Bernard Lyon 1, 69100 Villeurbanne, Franceemma.guillet@univ-lyon1.fr (E.G.-D.); 3 Maître de Conférences HDR, Université de Strasbourg, LISEC, 67000 Strasbourg, France; poparoch@unistra.fr

**Keywords:** well-being in childhood, well-being in adolescence, weight stigma, motivation for physical activity, self-esteem, life satisfaction

## Abstract

The purpose of the present study was to compare attitudes toward body weight and physical activity in both regular-weight and overweight/obese children and adolescents, and assessing relations between attitudes and self-esteem, motivation for physical activity, life satisfaction and level of physical activity. A total of 126 children (Mage = 12.2, SD = 3.4), divided into two subsamples (i.e., overweight/obese, N = 44, and regular-weight), voluntarily participated in the study. A series of univariate analyses of variance was conducted to examine the differences in the study variables across the subsamples. Correlational analyses were conducted to examine the relationships among the variables. The results indicated that obese/overweight participants expressed a more positive implicit attitude toward the thin category than regular-weight participants. Furthermore, among overweight/obese participants, implicit attitude toward physical activity was significantly negatively correlated with explicit attitude toward physical activity and general self-esteem. Significant differences between obese/overweight and regular-weight participants indicated that the status in terms of weight played a key role in attitudes toward the explored constructs.

## 1. Introduction

Obesity is a condition in which the amount of body fat exceeds the biological need of an individual and is viewed as a global epidemic affecting all age groups [1]. It is defined more precisely as the manifestation of a positive energy balance that has been sustained over an extended period. Excessive body weight is a serious public health concern in Western countries and is associated with negative consequences on social life and psychological well-being. In the present paper, we are interested in obese children and adolescents considered as a vulnerable social group [1,2,3]. The rise in obesity in children and adolescents is internationally recognized as a risk factor for their physical and psychological health [4]. Specifically, research revealed that obese children and adolescents are continually exposed to experiences that may adversely affect their self-esteem and life satisfaction [5]. In fact, overweight young people are recognized as a stigmatized social group [6,7,8] and they are negatively perceived as undesirable, unattractive, inadequate and devaluated by society standards. Being categorized as “fat” is often associated with negative social perception in Western societies in peers, teachers, family members or educators [9]. “Fat” children are negatively perceived, being labeled as “lazy”, “stupid”, “sloppy” or “dirty”. Moreover, they are seldom seen as “best friends” or “having lots of friends” [6]. Research showed that the relationship between overweight and lower self-concept in children and adolescents is mediated by parental criticism of weight and weight-based teasing from peers [10,11]. Also, among adolescents, weight-based teasing is linked to poorer self-esteem [2,12,13]. Overweight people seem to internalize the negative societal attitudes, and children’s attitudes toward obesity seem to emerge early in childhood [7]. Indeed, there is evidence that obese and regular-weight people report similar levels of dislike toward overweight people [14]. The existing literature suggests that weight stigma refers to social discrimination and devaluation of obese/overweight people [7,8,14,15]. According to Major [16], the process of weight stigma entails three major constructs: perceived discrimination (i.e., individual experiences of unfair treatment, linked to one’s weight), weight stigma concerns (i.e., individual anticipation of encountering weight-based avoidance and/or rejection) and weight bias internalization (i.e., self-directed weight stigma and self-devaluation, which leads to accepting the negative social stereotypes about weight). Moreover, weight bias is one of the latest acceptable forms of prejudice as the overweight individuals are considered to bear responsibility for the stigma they experience and because of the perception that stigma itself may motivate people to lose weight [14,15,17]. Therefore, obese children and adolescents who experience difficulties in a range of interpersonal contexts (e.g., teasing, social rejection, bullying) are likely to experience a decline in social status, and perceive themselves to be targets of prejudice and discrimination [3,7,18], which in turn negatively impacts their self-concept [12,19,20,21]. This would explain why obese children and adolescents consistently report experiencing lowered life satisfaction and self-esteem.

Obesity in childhood and adolescence is indeed a complex phenomenon, in which individual, relational and social mechanisms intersect [3]. Increasing understanding of the mechanisms involved in obesity during childhood and adolescence appears crucial to promote weight management strategies, and to facilitate adaptive health behavior. Accordingly, in the present study, we focused on physical activity (PA) as an adaptive health behavior involved in the prevention and management of obesity. Specifically, we were interested in increasing understanding of socio-cognitive predictors of PA in children and adolescents. PA is essential in the prevention of obesity in childhood and adolescence and plays an important role in healthy outcomes, such as life satisfaction or self-esteem [4,22,23]. Benefits of PA in childhood and adolescence are linked to the improvement in physical conditions involved in normal development and growth, and in the associated psychological mechanisms [2,24]. Despite the importance of PA for the healthful growth and development of children and adolescents, a large population of children and adolescents do not meet recommended PA guidelines [23]. Consequently, understanding the rationale behind the involvement in PA for children and adolescents is essential to help prevent obesity. In addition, focusing on children and adolescents is necessary to prevent the obesity-related complications in adulthood. Therefore, in our research, we were mainly interested in implicit and explicit attitudes and their role in health behavior such as PA, a topic that has received increasing interest in recent decades [25,26,27]. Explicit attitudes are defined as evaluative reactions toward social objects, which are self-reports and are vulnerable to social desirability concerns. On the other hand, implicit attitudes are conceptualized as evaluations that people cannot (because they are not aware of) or do not want to (because of social pressures) express overtly [28]. Importantly, these types of evaluations are, to some extent, predictive of the individuals’ behavior [29]. According to Olson and Fazio [30], explicit attitudes may orient deliberate behavior, while implicit attitudes may guide automatic and spontaneous behavior. From the health psychology perspective, the attitudes toward PA can be seen as crucial factors involved in obesity [31]. From this perspective, higher levels of PA are associated with implicit positive attitudes [32,33]. This kind of evidence suggests that implicit attitudes are linked with PA and that negative attitudes toward PA can be an important factor in predicting effective engagement in PA.

Other mechanisms can interfere with the effectiveness of weight management strategies and especially with the involvement in PA for children and adolescents. In this regard, weight bias is put into relation with decreased PA through the negative motivation for PA [15]. Thus, not only do negative social views impact adolescents’ self-perceptions but experiencing weight stigma increases the risk of engaging in unhealthy eating behaviors and lower levels of PA [14,18,34]. This evidence suggests that attitudes toward weight are also important mechanisms to consider in promoting health behavior, such as PA. To gain a more comprehensive view of the mechanisms involved in the phenomenon of obesity in childhood and adolescence and how to improve weight management strategies, it seems appropriate to focus simultaneously on attitudes toward PA and weight. As far as we know, previous studies have focused on attitudes toward PA or attitudes toward weight but have not taken them both into account.

Starting from these premises, the purpose of the present study was to investigate how young people’s attitudes toward body weight are linked to attitudes toward PA and psychological-related consequences. Specifically, this study aimed to compare attitudes toward body weight and PA in both regular-weight and overweight/obese children and adolescents. Moreover, we were interested in assessing relations between attitudes and psychological and physiological mechanisms, such as self-esteem, life satisfaction, motivation for PA and level of PA, in an exploratory manner.

## 2. Materials and Methods

### 2.1. Participants

A total of 126 children (57 girls and 60 boys, 9 participants did not specify their gender, Mage = 12.2, SD = 3.4) voluntarily participated in the study. It is noteworthy that 44 of them (29 girls, 15 boys, Mage = 10.9, SD = 3.05) were diagnosed by a family doctor or a pediatrician as being overweight or obese, according to the International Obesity Taskforce sex- and age-specific cut-off values for BMI [35]. As such, overweight and obese children were followed by a network of health professionals providing territorial coordination of care (multidisciplinary, personalized and local care). Indeed, they were regularly followed by different healthcare providers specializing in treating obese or overweight people, including doctors, dieticians, psychologists, physiotherapists and teachers in adapted PA. Specifically obese/overweight participants were enrolled in a structured PA program including effective and regular exercise (e.g., Nordic walking, hiking, group games, swimming), supported by health messages about the importance of PA. The PA program was planned throughout the school year (from 8 to 10 months depending on the date of entry into the program), included two PA sessions per week and was implemented by an adapted PA teacher. The focus of the program was to show that PA is accessible to obese/overweight people. Thus, program participants practiced and also discussed nutrition. The idea was to develop autonomy in PA practice once the program ended.

### 2.2. Measures

**Figure 1:**
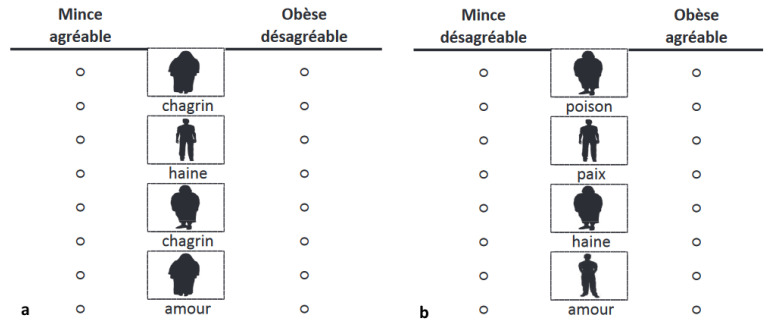
Extract from congruent (**a**) and incongruent (**b**) Thin–Obese IAT block.

**Figure 2:**
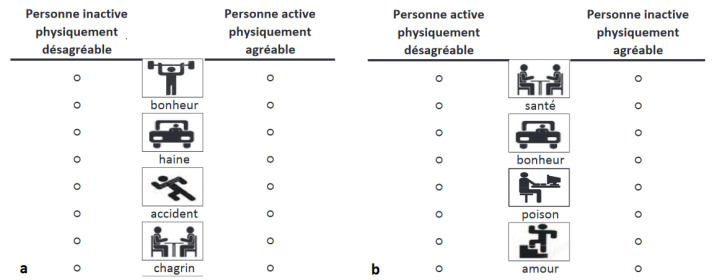
Extract from congruent (**a**) and incongruent (**b**) Physical Activity–Physical Inactivity IAT block.

To measure implicit attitudes toward bodyweight and PA, an Implicit Association Test (IAT) published by Greenwald et al. [36,37] was used in its paper–pencil version [38]. The IAT measures attitudes (affective reactions) toward social objects (for a recent review, see Epifania et al. [39]). Given their social significance, obesity and PA are social objects that can be used in an IAT. An IAT assesses the strength of associations between two contrasting attitude objects (e.g., thin and obese in a Thin–Obese IAT) and two evaluative dimensions (pleasant and unpleasant attributes). It records participant’s reaction times to two combined categorical arrangements (i.e., congruent vs. incongruent). For the congruent phase of the Thin–Obese IAT, the categories Obese and Unpleasant, first, and Thin and Pleasant, second, are combined (see Figure 1). For the incongruent phase, the categories Obese and Pleasant, on the one hand, and Thin and Unpleasant, on the other hand, are joined. For the Physical Activity–Physical Inactivity IAT, the congruent phase combines the categories Physical Inactivity and Unpleasant, on the one hand, and Physical Activity and Pleasant, on the other hand. The incongruent phase combines the categories Physical Inactivity and Pleasant, and Physical Activity and Unpleasant (see Figure 2).

The incongruent phase is significant of an existing bias in the participant toward one of the two categories; therefore, the congruent phase is typically finished faster [36]. In the present study, each participant completed three IATs. They started with a Flower–Insect training IAT in a paper–pencil version that allowed participants to familiarize themselves with the procedure before the critical IATs. In the research-relevant IATs, the target categories Obese–Thin, on the one hand, and Physical Activity–Physical Inactivity, on the other hand, were represented by four pictograms. The pilot studies showed that they are clearly associated with one of the two target categories. Each of the two evaluative categories, Pleasant and Unpleasant, was represented by four words (happiness, love, peace and health, for Pleasant, and accident grief, poison and hate, for Unpleasant). Each IAT task comprised two pages, one for the congruent phase and one for the incongruent phase. Each page was split into two matching vertical blocks. On the upper part of each block, the target categories and the evaluative categories (but also the exemplars representing them) were displayed. A circle was drawn on both sides of a given item (right and left).

The middle column of each page comprised 24 items from the four categories, pictograms and words, displayed alternately, that is, a word, a pictogram, a word, a pictogram, etc. Participants were encouraged to complete the task as quickly as possible and to avoid mistakes or revising mistakes. They had 20 s to perform as many categorizations as possible and had to categorize the stimuli, one after the other, by ticking a cross in one of the circles on either side of each word. They started by categorizing the first word in the left block. When they reached the end of the left block, they continued with the first word in the right block. An audible signal indicated the beginning of the task. After 20 s, the participants stopped and marked the last word they had processed. They continued with the second page. The experimenter added that the categories were identical, the only change was that their position was reversed. For each IAT page, the number of correct responses was counted. The algorithm proposed by Nosek [40] for paper–pencil versions enabled us to calculate the IAT score as follows:

$$\frac{\pm \;maximum\;value[A,B]}{minimum\;value[A,B]}\sqrt{\left|(A-B)\right|}$$

where *A* and *B* represent the number of correct answers in the two IAT blocks (e.g., *A* = compatible phase, *B* = incompatible phase). The higher the score, the more negative the implicit attitudes toward obesity, on one hand, and physical inactivity, on the other hand.

The alpha coefficients for the two composite scores showed that the reliability was acceptable: implicit attitudes toward obesity (α = 0.70), and implicit attitudes toward PA (α = 0.69).

Explicit attitudes toward body weight and PA were measured through a semantic differentiator. Concerning the categories Obese and Thin, participants rated each of them on seven bipolar dimensions (e.g., ugly vs. beautiful; nasty vs. kind; unpleasant vs. pleasant). The Physical Activity and Physical Inactivity categories were also rated on seven semantic differentiators (e.g., beneficial vs. harmful; interesting vs. annoying). Each item was rated on a 7-point scale ranging from 1 (negative) to 7 (positive). The difference scores between the ratings of the two categories were calculated to evaluate attitudes toward obese people relative to thin people, on the one hand, and toward physical activity relative to physical inactivity. Higher scores indicated bias toward obese and physical activity, respectively. The reliability for the two composite scores was acceptable according to the alpha coefficients: explicit attitudes toward obesity (α = 0.75), and explicit attitudes toward PA (α = 0.88).

To evaluate the participants’ motivation for PA, the French version of the behavioral regulation in exercise questionnaire-3 (BREQ-3; [41,42] was used. Drawing from self-determination theory [43], the BREQ-3 comprises six four-item subscales measuring intrinsic motivation; integrated, identified, introjected and external regulations; and amotivation. To assess autonomous motivation (the mean of intrinsic motivation, integrated regulation and identified regulation) and controlled motivation (the mean of introjected and external regulations), two composite scores were calculated According to the alpha coefficients, the reliability was acceptable for the two composite scores: autonomous motivation (α = 0.88), and controlled motivation (α = 0.67).

To assess life satisfaction, the French version [44] of the satisfaction with life scale (SWLS; [45]) was employed. Participants had to report their agreement with the five items of the scale from 1 (does not correspond at all) to 7 (corresponds very strongly). The alpha coefficient showed an acceptable reliability was acceptable according to the alpha coefficient of 0.71.

The French physical self-inventory in its short version (PSI; [46,47]) was used to measure self-esteem. This self-report questionnaire comprises six subscales, with two items each corresponding to general self-esteem, physical condition, physical self-worth, physical attractiveness, sport competence and physical strength. Cronbach’s alpha does not appear to be appropriate for two-item scales as it depends on the number of items of a scale [48]. In these cases, item analysis is more suitable to estimate the internal reliability of scales [48,49]. Therefore, for each PSI item, the following criteria were applied [49]: (a) a minimum item–total correlation coefficient of r = 0.40 and (b) an inter-item correlation between 0.20 and 0.70. The 12 items reached all the criteria, indicating acceptable PSI reliability. All PSI items were assessed on a six-point Likert scale from 1 (completely disagree) to 6 (completely agree).

The French version of the short International Physical Activity Questionnaire for adolescents (IPAQ-A; [50]) was used to assess adolescents’ levels of PA for the participants who were 12 years old and older. The adolescents were asked to report the amount of PA performed during the 7 days prior to the questionnaire being filled in. The duration (minutes/day) and the frequency (number of days per week) of PA practice for walking, moderate PA and vigorous PA were assessed. Walking activity encompassed overall walking (at home, during free time, at school, sport or recreation, to get from one place to another). Moderate PA included activities that imply moderate physical effort and require harder-than-normal breath (e.g., sweeping, carrying light loads). Vigorous PA comprised activities that require hard physical effort and make the adolescent breath much harder than normal (e.g., mountain biking, playing football, digging in the garden or yard). Based on the guidelines for analysis of the IPAQ and data processing, an average MET (in metabolic equivalent) score was computed for each type of activity: 3.3 for walking, 4.0 for moderate activity and 8.0 for vigorous activity. A MET-minute score was then calculated by multiplying the MET score by the minutes performed, giving energy expenditure (in metabolic equivalent (MET)-minutes per week) and for each type of activity. Finally, the total energy expenditure was calculated as the sum of vigorous PA, moderate PA and walking MET-min per week scores [50].

Because the IPAQ-A was not adapted for children under 12 years old, the levels of PA for the participants who were 11 years old and younger were assessed using a PA questionnaire for school children (QAPE-semaine; [51]). The QAPE-semaine measures physical activities at school, during leisure-time and other activities. This questionnaire is constructed in three parts: (a) PA at school last week (school courses of physical education and sport, recreation, school trips); (b) PA last week outside of school (a list of different sport and physical activities from the sports classification proposed by the French Ministry of Youth, Sports and Community Life); and (c) other PA from last week designed to assess the intensity (not at all, a little and very active) of activities practiced outside the school (which were specified within the aforementioned list) and the number of moments spent in front of a screen (e.g., television, video games). As a whole, three scores were calculated using the QAPE-semaine: variety of PA, intensity of PA and sedentary.

### 2.3. Procedure

The research followed American Psychological Association (APA) international ethical guidelines. Parental consent was obtained, as well as the educational authorities’ authorization to enroll children and students in the study. Written informed consent was obtained from participants prior to data collection. The research was conducted in groups of 5–15 participants and one of the research team members provided instructions.

### 2.4. Data Analysis

First, univariate analyses of variance (ANOVAs) were performed to test whether the differences between the two subsamples of the study (i.e., overweight/obese versus regular-weight) were detected across the variables stake in our study. The effect size was estimated with partial eta squared (η^2^). Second, the relations among the measures of the different variable were assessed through correlational analyses, i.e., implicit and explicit attitudes toward PA and toward thin–obese with self-esteem, motivation for PA, life satisfaction and level of PA. Correlational analyses (using Pearson correlations) were conducted separately for the two subsamples of the study to explore whether obesity/overweight influenced the relationships between variables in our study. Effect sizes are considered as small (0.1 < r < 0.3), moderate (0.3 < r < 0.5) or large (0.5 < r) [52] as a function of the r value. For both ANOVAs and correlational analyses, the significance threshold was set at *p* equal to 0.05 and *p* values ranging from 0.05 to 0.08 were considered as marginally significant.

## 3. Results

### 3.1. Subsample Differences in Study Variable Scores

The results of the ANOVAs are presented in Table 1. The results revealed that overweight/obese participants reported significantly higher scores of explicit attitude toward PA, implicit attitude toward thin–obese and level of PA (IPAQ-A scores) than regular-weight participants. In contrast, regular-weight participants reported significantly higher scores of general self-esteem and sport competence than overweight/obese participants.

### 3.2. Correlational Analyses across Subsamples

The results of the correlational analyses are presented in Table 2. Some correlations were significant (or marginal with a *p* ≤ 0.08) for both subsamples. Especially, explicit attitude toward PA was positively moderately correlated to autonomous motivation (r = 0.38 and 0.44 for overweight/obese and regular-weight participants, respectively), whereas explicit attitude toward thin–obese was positively moderately correlated with sport competence (r = 0.41 and 0.32) and physical attractiveness (r = 0.28 and 0.33). Other correlations were significant (or marginal with a *p* ≤ 0.08) in only one subsample. Indeed, among the overweight/obese participants, implicit attitude toward PA was negatively correlated with explicit attitude toward PA (r = −0.27, low effect size), general self-esteem (r = −0.33, moderate effect size) and sport competence (r = −0.40, moderate effect size), and positively moderately correlated with level of PA (r = 0.47). Similarly, explicit attitude toward PA was positively moderately correlated with explicit attitude toward thin–obese (r = 0.43), general self-esteem (r = 0.36), sport competence (r = 0.35) and physical attractiveness (r = 0.35), whereas explicit attitude toward thin–obese was moderately correlated with autonomous motivation (r = 0.29), physical condition (r = 0.40) and sedentary (r = −0.37). In contrast, several correlations were only significant among the regular-weight participants. Indeed, implicit attitude toward PA was positively strongly correlated with implicit (r = 0.60) and explicit (r = 0.23, low effect size) attitude toward thin–obese, whereas explicit attitude toward PA was weakly correlated with controlled motivation (r = 0.22) and physical condition (r = 0.28). Similarly, explicit attitude toward thin–obese was weakly correlated with implicit attitude toward thin–obese (r = 0.20), satisfaction with life (r = 0.22), general self-esteem (r = 0.23) and level of PA (r = −0.43, moderate effect size).

## 4. Discussion

This study aimed to compare attitudes toward PA and body weight in regular-weight, on the one hand, and overweight/obese children and adolescents, on the other. Moreover, we were interested in assessing relations between attitudes and constructs implied in PA practice such as motivation, life satisfaction and self-esteem, as well as with self-reported levels of PA. The main findings of this study concern two issues. Firstly, the significant differences between obese/overweight and normal-weight participants indicated the importance of weight status with regard to attitudes toward the explored constructs. Secondly, our results supported the interest in simultaneously considering implicit and explicit attitudes toward PA and weight to advance understanding of the mechanisms responsible for children and adolescents’ participation in PA.

The results indicated that explicit attitudes toward PA, but not implicit attitudes, were more positive in obese/overweight than in regular-weight participants. As previously mentioned, the obese/overweight participants were enrolled in a program including effective and regular exercise. In this program, they also were made aware of the importance of PA and they were exposed to health messages, which might have improved self-reported explicit attitudes toward PA but not implicit ones. The same tendency is present for implicit attitudes toward weight, namely, obese/overweight participants expressed a preference toward thin relative to obese, therefore holding more positive attitudes toward the thin category than regular-weight participants. This is consistent with the literature showing that some stigmatized social groups express negative attitudes toward their own group, which is making them even more vulnerable [7,8,14]. These attitudes could be seen as the result of a process of identity construction influenced by the weight stigmatization experienced from an early age. The sources of the weight stigmatization include family entourage, reference adults such as teachers and peer group which could lead obese/overweight people to stigmatize members of their group in turn [4]. Among obese/overweight children and adolescents, weight stigma is experienced in the form of victimization, such as bullying or teasing, and weight bias internalization is linked to poor weight-related health [16,18,20]. Interestingly, in the present study, obese/overweight participants did not express bias at an explicit level. A motivation to project a non-biased image of themselves might explain this result. Indeed, it has been shown that weight bias internalization enforces social norms: by conforming to the prevailing expectations of society, stigmatized people could be accepted back as “valued” members [6]. Furthermore, the health messages received in the program, which aimed to enhance qualities and facilitate access to PA, may have encouraged explicit belief in the absence of the bias. The elements above suggest the importance of considering implicit and explicit attitudes simultaneously in order to have a better understanding of how to support PA participation. As far as general self-esteem and sport competence are concerned, regular-weight participants expressed higher levels than obese/overweight participants. On the other hand, obese/overweight participants self-reported high levels of PA, greater than the ones reported by regular-weight participants. It is important to point out that in the program in which the obese/overweight participants were involved, health messages emphasized PA as something accessible to all, as opposed to sport, which would imply specific skills, which can explain these data.

Some relations among variables are to be mentioned. In obese/overweight participants, implicit and explicit attitudes toward PA were negatively correlated. This negative relationship within the two levels of attitudes can be explained by taking into account the fact that among the stigmatized public, health messages touch the explicit level but do not profoundly change reactions. There could thus be a dissociation between the implicit and explicit attitudes toward PA. In contrast, the regular-weight population feels less concerned by health messages provided by the school or the media. Consistently, explicit attitudes toward PA were positively associated with explicit attitudes toward weight for obese/overweight participants but not for the regular-weight population. Furthermore, implicit attitudes toward PA were positively associated with implicit attitudes toward weight for regular-weight but not for obese/overweight participants. This supports the fact that obese/overweight people internalize at an implicit level the social valorization of thinness, which in turn is associated with the valorization of exercise. The articulation between attitude toward PA and attitude toward weight remains a complex process. Research suggested that weight stigma inhibits PA behaviors [8,34]. On the other hand, recent studies suggested that increasing access to a larger variety of PA reinforcement, allowing free choice, should improve PA practice, developing preferences for healthy behaviors [22]. In addition, the processes through which weight stigma influences the practice of PA deserve further investigation. It has been shown that people who have internalized the weight stigma are more motivated to avoid PA. When this is combined with poor self-control resources, it can result in lower PA [15]. However, in the present study, explicit PA attitudes were similarly correlated with the motivational variables in both groups. Specifically, explicit PA attitudes were positively associated with both the autonomous and controlled forms of motivation for PA. Considering that explicit attitudes are evaluative reactions vulnerable to social desirability concerns, these processes could be investigated in the future. Finally, in obese/overweight participants, implicit PA attitudes were negatively associated with general self-esteem, whereas explicit attitudes were positively associated with it. The same is true for perceptions of sports competence. This is not the case in the regular-weight population where all these correlations were non-significant. This finding supports the importance of implicit attitudes in positive health outcomes [25,26,27].

From a theoretical point of view, the results of the present study offer insights into the differential impact of the status in terms of weight (i.e., obese/overweight and regular-weight) on attitudes toward weight and PA but also on some other construct shown to be implied in exercise. The simultaneous consideration of implicit and explicit attitudes toward PA and weight is a strength of the study. Significant differences emerged between obese/overweight and regular-weight participants which indicated that status in terms of weight could play a key role in attitudes toward weight and PA. These elements contribute to the literature on implicit and explicit attitudes toward health in children and adolescents, especially regarding the very critical area of obesity [2,3]. The present study also adds to the general literature on implicit attitudes toward sensitive social objects in order to elucidate how individuals affectively react to them despite desirability issues. Moreover, it indicates how these attitudes are related to other significant socio-cognitive factors in order to better predict adaptive health behavior in children and adolescents. It is important to take into account that the present study was conducted in a population that is difficult to reach. During childhood and adolescence, self-esteem is changeable, uncertain and under construction. At the same time, this is a population potentially receptive to positive health messages. Understanding the factors that lead to PA in childhood and adolescence would enhance a positive spiral of appropriate health behaviors.

Some limitations of the study must be mentioned. While the fact that the participants were involved in a program made it easier to reach a population of obese/overweight children and adolescents, it also makes it difficult to generalize the results. There is also an imbalance in the sample with regard to gender, with an overrepresentation of girls. The analysis of the data, consistent with the exploratory purpose of the study, does not allow us to go any further into the complexity of the relationships among the observed variables. Further analysis will be needed to complement the results already obtained. Research is clearly needed to examine these relations with diversified samples. In future studies, the control group of regular-weight children and adolescents should also benefit from an awareness-raising program. This element seems difficult to achieve as public policies invest more in vulnerable populations, such as people with chronic or long-term illnesses. Despite its limitations, this study provides new knowledge to understand the processes at work in attitudes toward PA and body weight during childhood and adolescence.

The practical implications of such an understanding would allow the construction and evaluation of PA obesity prevention. This study suggests that simultaneously taking into account implicit and explicit attitudes toward PA and weight seems crucial to sustain participation in PA programs. Firstly, the results of the study support the importance of raising awareness of the difference between sport and PA. Designing awareness programs that circulate health messages aimed at valuing PA as something accessible to all could support the development of positive explicit attitudes toward PA. Explicit attitudes toward physical activity might foster positive representations of practice during childhood and adolescence, both for children/adolescents and their parents. As we have seen though, health messages do not profoundly touch the implicit level and are not enough to change reactions. Our results suggest that it is more complex to succeed in changing implicit attitudes. In this sense, it seems important to consider the relationship between negative implicit attitudes toward PA and weight and experiences of weight stigmatization. In fact, in order to obtain the physical and psychological benefits associated with the practice of PA, the experience of PA must be perceived as psychologically safe by children and adolescents. Therefore, the content of the PA sessions should take into account any previous weight stigmatization experienced by obese/overweight children and adolescents. PA should be seen as the setting to overcome stereotypes and weight bias internalization. To achieve this, PA programs for obesity prevention should be designed by sports professionals in collaboration with health professionals. It also seems necessary to involve and raise awareness among members of all relational systems involved in the lives of children and adolescents, such as families, schools, peer groups, educators and health care institutions. In fact, it would be appropriate to act on explicit and implicit attitudes toward PA and weight of parents and adults involved in caring, in order to produce more profound and lasting social and political changes.

## Figures and Tables

**Table 1:** Subsample differences in study variable scores.

	Overweight–Obese		Regular-Weight				
	*M*	*SD*	*M*	*SD*	*F*	*p*	*η^2^*
Implicit attitude toward physical activity	1.03	6.63	0.28	5.24	0.06	0.812	0.00
Explicit attitude toward physical activity	3.39	1.86	2.24	2.52	7.46	0.007	0.06
Implicit attitude toward thin–obese	2.16	3.64	−0.33	6.25	5.84	0.017	0.05
Explicit attitude toward thin–obese	0.56	1.53	0.42	0.98	0.43	0.512	0.00
Controlled motivation	1.71	0.95	1.45	0.65	1.90	0.171	0.02
Autonomous motivation	2.89	0.66	2.99	0.83	0.60	0.440	0.00
Satisfaction with life	4.96	1.34	5.12	1.12	0.16	0.686	0.00
General self-esteem	3.41	1.60	4.12	1.32	5.16	0.025	0.04
Physical self-worth	4.23	1.32	4.09	1.21	0.28	0.598	0.00
Physical condition	3.92	1.22	4.02	1.27	0.23	0.631	0.00
Sport competence	3.41	1.53	4.00	1.41	4.07	0.046	0.03
Physical attractiveness	3.63	1.28	3.73	1.05	0.02	0.879	0.00
Physical strength	3.88	1.15	3.84	1.09	0.06	0.811	0.00
Physical activity level (IPAQ-A)	4438.95	1814.60	130.38	55.36	107.18	<0.001	0.75
Physical activity variability (QAPE)	16.81	5.69	14.35	5.32	3.18	0.079	0.05
Physical activity intensity (QAPE)	3.38	3.07	3.60	1.82	0.13	0.722	0.00
Sedentary (QAPE)	10.27	7.78	8.65	5.55	0.97	0.328	0.01

Note. IPAQ-A International Physical Activity Questionnaire for adolescents; QAPE Questionnaire de Mesure de l’activité Physique Chez l’enfant.

**Table 2:** Correlational analyses across subsamples.

	1	2	3	4
1. Implicit attitude toward physical activity	–			
2. Explicit attitude toward physical activity	−0.27 ^¥^/−0.16 ^a^	–		
3. Implicit attitude toward thin–obese	0.02/0.60 *	0.08/−0.14	–	
4. Explicit attitude toward thin–obese	0.06/0.23 *	0.43 */−0.02	0.26/0.20 ^¥^	–
Controlled motivation	−0.07/−0.17	0.15/0.22 *	−0.16/−0.18	0.13/−0.10
Autonomous motivation	−0.01/0.01	0.38 */0.44 *	−0.21/−0.02	0.29 ^¥^/−0.09
Satisfaction with life	0.00/−0.02	0.03/0.12	−0.03/0.04	0.03/0.22 *
General self-esteem	−0.33 */−0.03	0.36 */0.01	0.17/−0.12	0.20/0.23 *
Physical self-worth	−0.13/0.09	0.26/0.05	0.24/0.03	0.06/0.17
Physical condition	−0.18/0.05	0.23/0.28 *	−0.02/−0.04	0.40 */0.13
Sport competence	−0.40 */0.02	0.35 */0.09	0.07/−0.04	0.41 */0.32 *
Physical attractiveness	−0.13/0.01	0.35 */0.06	0.22/−0.04	0.28 ^¥^/0.33 *
Physical strength	−0.23/0.05	0.06/0.15	−0.02/0.03	0.07/0.01
Physical activity level (IPAQ-A)	0.47 ^¥^/0.08	0.02/0.34	0.29/0.19	0.18/−0.43 ^¥^
Physical activity variability (QAPE)	−0.01/0.00	0.32/−0.25	0.11/0.14	0.28/0.12
Physical activity intensity (QAPE)	0.04/0.18	0.22/−0.08	−0.13/0.19	0.06/0.03
Sedentary (QAPE)	−0.13/0.13	0.29/−0.07	−0.05/0.26	−0.37 ^¥^/0.11

Note. ^a^ correlation for the obese/overweight subsample/correlation for the control subsample; * *p* < 0.05 ^¥^ *p* < 0.09; IPAQ-A International Physical Activity Questionnaire for adolescents; QAPE Questionnaire de Mesure de l’activité Physique Chez l’enfant.

## Data Availability

The data presented in this study are available on request from the corresponding author.
